# Cultivar Evaluation and Essential Test Locations Identification for Sugarcane Breeding in China

**DOI:** 10.1155/2014/302753

**Published:** 2014-05-20

**Authors:** Jun Luo, Yong-Bao Pan, Liping Xu, Hua Zhang, Zhaonian Yuan, Zuhu Deng, Rukai Chen, Youxiong Que

**Affiliations:** ^1^Key Laboratory of Sugarcane Biology and Genetic Breeding, Ministry of Agriculture, Fujian Agriculture and Forestry University, Fuzhou 350002, China; ^2^USDA-ARS, Sugarcane Research Laboratory, Houma, LA 70360, USA

## Abstract

The discrepancies across test sites and years, along with the interaction between cultivar and environment, make it difficult to accurately evaluate the differences of the sugarcane cultivars. Using a genotype main effect plus genotype-environment interaction (GGE) Biplot software, the yield performance data of seven sugarcane cultivars in the 8th Chinese National Sugarcane Regional Tests were analyzed to identify cultivars recommended for commercial release. Fn38 produced a high and stable sugar yield. Gn02-70 had the lowest cane yield with high stability. Yz06-407 was a high cane yield cultivar with poor stability in sugar yield. Yz05-51 and Lc03-1137 had an unstable cane yield but relatively high sugar yield. Fn39 produced stable high sugar yield with low and unstable cane production. Significantly different sugar and cane yields were observed across seasons due to strong cultivar-environment interactions. Three areas, Guangxi Chongzuo, Guangxi Baise, and Guangxi Hechi, showed better representativeness of cane yield and sugar content than the other four areas. On the other hand, the areas Guangxi Chongzuo, Yunnan Lincang, and Yunnan Baoshan showed strong discrimination ability, while the areas Guangxi Hechi and Guangxi Liuzhou showed poor discrimination ability. This study provides a reference for cultivar evaluation and essential test locations identification for sugarcane breeding in China.

## 1. Introduction

Sugarcane (*Saccharum *spp. hybrids) is an important sugar crop in China. Its planting acreage accounted for 92% of the total sugar crops and reached 1.586 million ha in 2012. The main areas of sugarcane production located in the south and central parts of Guangxi Province, southwest part of Yunnan Province, west part of Guangdong Province, and north part of Hainan Province. The ROC (People's Republic of China) cultivars from China Taiwan account for more than 80% of the total sugarcane planting area in Mainland China, resulting in a short harvesting season with low average sugar yield and serious pests in large area [1]. Therefore, sugarcane breeding and cultivar distribution should be accelerated to achieve environment suitable cultivations of multiple cultivars with different maturity date (early, intermediate, and late). In regional trials of cultivars, not only the yielding ability and stability of the sugarcane cultivars are evaluated, but also new cultivars suitable to specific areas may be identified, leading to the multicultivar distribution [1–4]. However, during the tests, the discrepancies across test sites and years, along with the interaction between cultivar and environment, make it difficult to accurately evaluate the differences of the sugarcane cultivars [1–3]. Thus, it is vital to find out a proper program to statistically analyze the data to avoid biased evaluation of cultivars [5, 6].

The additive main effect and multiplicative interaction (AMMI) model has been widely used for analyzing the multilocation varietal trials in many crops, namely,* Triticum aestivum* [7, 8],* Oryza sativa* [9],* Brassica napus* [10], and sugarcane [11]. However, the AMMI model relies on two-way data, which tends to overlook some high yielding but poor stability cultivars or ones with high stability but low yielding [12, 13]. Previous studies revealed that genotype × environment (*G* × *E*) interaction and yield stability can be analyzed under different environmental conditions by a new method called genotype main effect plus genotype-environment interaction (GGE) Biplot analysis [14–25]. This model focuses on both effects of genotype (*G*) and (*G* × *E*), where the data was treated with environment centralization. The GGE-Biplot method has been proven to be a useful tool for data from multiple sites/years [14–17] on many crop species, including* Lactuca sativa* [18],* Arachis hypogaea* [19],* B. napus* [20],* Glycine max* [21],* T. aestivum* [22],* Hibiscus mutabilis* [23], and* Helianthus annuus* [24]. The GGE-Biplot method has also been used in sugarcane to analyze and identify the high and stable yielding cultivars [2, 25]. However, it has not yet been used in the data analysis from National Sugarcane Varietal Regional Tests in China.

Unlike other crops, data from ratoon crops are also required from Regional Tests in sugarcane. Compared to the plant cane crop, the ratoon crops are affected more by the environments. As such, the (*G* × *E*) interaction has a greater impact on the analysis on yield stability and adaptability [11], especially on the three most desirable traits, namely, cane yield, sugar yield, and stability. In this paper,we aim to demonstrate a simple and effective method for analyzing data from the 8th Chinese National Sugarcane Regional Tests.

## 2. Materials and Methods

### 2.1. Cultivars and Test Sites

Six cultivars, namely, Funong 38 (Fn38), Funong 39 (Fn39), Yunzhe 06-407 (Yz06-407), Yunzhe 05-51 (Yz05-51), Liucheng 03-1137 (Lc03-1137), and Gannan 02-70 (Gn02-70), and one control (Roc22) were involved in the study. Seven experimental sites within the major sugarcane planting areas were selected for representativeness analysis, including Guangxi Liuzhou (GXLZ; E 109°22′, N 24°28′; altitude 99.1 m; yellow soil), Guangxi Chongzuo (GXCZ; E 108°32′, N 22°56′; altitude 78 m; loam soil), Guangxi Laibin (GXLB; E 109°05′, N 23°46′; altitude 95 m; sandy soil), Guangxi Baise (GXBS; E 106°98′, N 23°68′; altitude 82.5 m; sandy soil), Guangxi Hechi (GXHC; E 108°05′, N 24°05′; altitude 110 m; red loam), Yunnan Baoshan (YNBS; E 98°89′, N 24°91′; altitude 670 m; sandy soil), and Yunnan Lincang (YNLC; E 99°57′, N 24°05′; altitude 1030 m; red loam).

### 2.2. Experimental Design and Data Collection

Plant cane trials were conducted in 2011, and the first ratoon crop trials were conducted in 2012. The field trials used a triplicated randomized block design. There were four rows for each cultivar, with the amount of 10,500 sugarcane two-bud sets per ha. The plot area was 33.0 m^2^, with the row length of 7.5 m and the row space of 1.1 m. Cultural practices (intertill hilling, fertilization, irrigation, and pest control) were carried out on the same day for the same site. Data were collected on plant height, stalk diameter, number of stalks, and stalk weight. Sugarcane plants in the middle row in each plot were all harvested and weighed. The areas of sampled sugarcane were measured. The number of millable stalks within the sampling area was also counted. The single stalk yield and cane yield were calculated by the following formula:

(1) Number  of  millable  stalks  per  hectare  (number/hectare)=[(Number  of  millable  stalks  at  sampling  site  (number))×(area  of  sampling  site  (square  meters))−1]×10000,Single  stalk  yield=Height×(stalk  diameter)2×0.7851000Cane  yield  (kg/hectare)=[(Cane  yield  at  sampling  site  (Mg)Number  of  stalks  (number))×(Number  of  stalks  per  hectare  (number/hectare))] ×(1000)−1.

At the middle of each month from November through March, six healthy stalks, including five main stalks and one tiller stalk, were sampled for the measurement of sucrose content. Sucrose content was determined on a polarimeter (AP-100, ATAGO Co. Ltd., Japan). The average sugar yield per hectare was calculated based on the monthly average cane yield per hectare and monthly average sucrose content as follows:

(2) Sugar  yield  per  hectare  (Mg)=  [Cane  yield  per  hectare  (kg)×  sucrose  content  (%)]1000.

### 2.3. Data Processing

GGE-Biplot software [15] was adopted for data processing. Yield trait data were first filled into a two-way (cultivar-site) table, where each value (*P*) represented the mean of the corresponding cultivar at one particular test site. *P* = *M* + *E* + *G* + GE, where *M* represented total average value of a particular trait from multisite trials, *E* the main effect of the environment, *G* the main effect of the cultivar, and GE the genotype-environment interaction. A new genotype-environment two-way table was then generated by subtracting the sum of *M* and *E* from each value in the original two-way table. Since only *G* and GE were involved in the environmentally centralized genotype-environment two-way table, this cultivar evaluation tool was named as GGE-Biplot [16]. A correlation between principal and binormal vectors was estimated by the cosine value of the corresponding angle in the figure. Correlation coefficient of any two vectors was estimated by using the cosine value of the angle between the two vectors in the figure with one vector being assigned as start. Average cultivar performance and site representativeness were assessed by the position of cultivar (pilot) projection on the AT axis (average-tester axis). Yield stability and site discrimination ability were assessed by the projection length of the cultivar (pilot) on the AT axis [15]. Average value of each trait was calculated using the DPS software [26].

## 3. Results

### 3.1. Yield Performance and Variance Analysis

For the 2011 plant cane trials, Yz06-407 produced the highest cane yield of 119.30 Mg·ha^−1^, which was 12.61% higher than the control Roc22 (Table 1). Fn38 ranked the second at 115.61 Mg·ha^−1^, 9.12% higher than Roc22. The third rank was Yz05-51 at 111.58 Mg·ha^−1^, 5.32% higher than Roc22. The cane yields of Lc03-1137 and Fn39 were equal to that of Roc22. For the 2012 ratoon crop test, Yz06-407 again topped the list with a cane yield of 118.12 Mg·ha^−1^, which was 22.98% higher than the control. Fn38 again ranked the second (109.50 Mg·ha^−1^) and yielded 14.00% more than the control. Fn39 and Gn02-70 had average cane yields of 96.46 Mg·ha^−1^ and 93.56 Mg·ha^−1^, respectively, which were equal to the cane yield of Roc22.

For the 2011 plant cane trials, Fn38 had the highest sugar yield of 17.23 Mg·ha^−1^, which was 11.29% higher than Roc22 (Table 1). Yz05-51 had a yield of 16.76 Mg·ha^−1^, which was increased by 8.26% compared with that of the control. The lowest sugar yields were produced by Gn02-70 (15.46 Mg·ha^−1^) and Yz06-407 (15.59 Mg·ha^−1^), which were equal to that of Roc22. For the 2012 ratoon crop test, Fn38 again had the highest sugar yield of 17.16 Mg·ha^−1^, 16.75% more than Roc22. The sugar yields for Yz06-407, Yz05-51, and Lc03-1137 were 16.56 Mg·ha^−1^, 16.43 Mg·ha^−1^, and 16.22 Mg·ha^−1^, respectively, which were 12.67%, 11.74%, and 10.32% higher than Roc22, respectively (Table 1).

The estimates of variance components are presented in Table 2. The largest proportion of the total variation in environment was accounted for by the main effect of trials followed by the (*G* × *E*) interaction component and then the genotype. This highlights the importance of trial site effects on the (*G* × *E*) interaction and, at the same time, suggests that only a small proportion of the total variation was due to the mean differences between cultivars and that the genotype × environment interaction was more pronounced.

### 3.2. The Most Adaptive Sites

#### 3.2.1. Cane Yield

**Figure 1:**
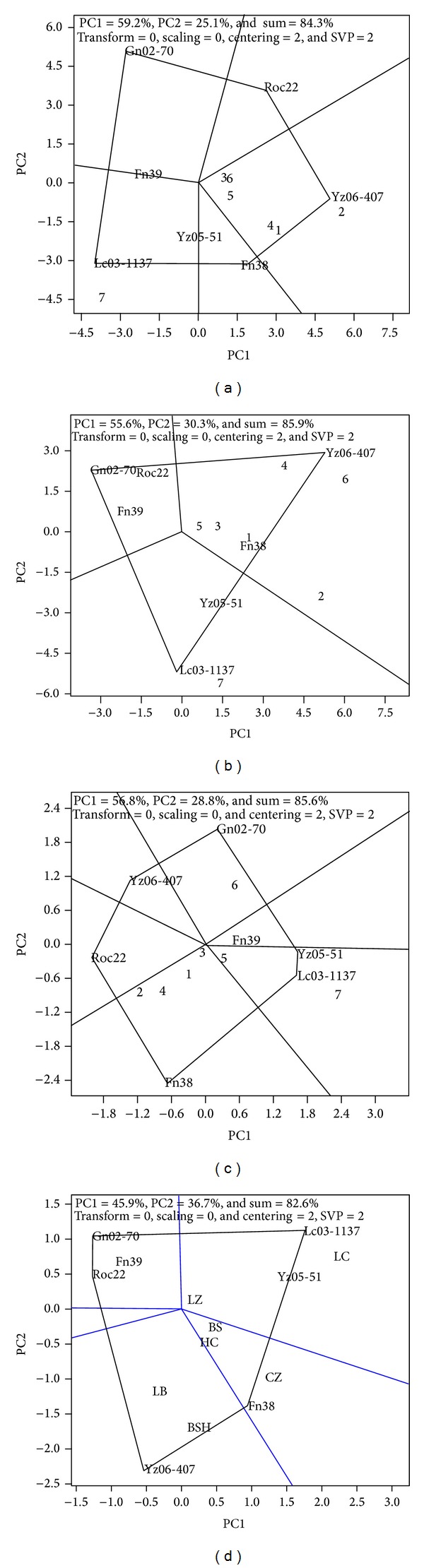
Adaptability of sugarcane cultivars based on GGE-Biplot analysis. Cultivars with the longest distance are linked to the original point to form a multilateral figure and vertical lines are drawn to each side of the figure to divide the whole biplot into several fan-shaped regions and to assort evaluation sites into different groups. Within each group, the cultivars located at the apex of the multilateral figure represent the best ones among the cultivars within the fan-shaped region: (a) 2011 plant crop cane yield; (b) 2012 ratoon crop cane yield; (c) 2011 plant crop sugar yield; and (d) 2012 ratoon crop sugar yield. PC1 = principal component 1; PC2 = principal component 2. Numerical codes for evaluation Sites: 1 = Guangxi Baise; 2 = Guangxi Chongzuo; 3 = Guangxi Hechi; 4 = Guangxi Laibin; 5 = Guangxi Liuzhou; 6 = Yunnan Baoshan; and 7 = Yunnan Lincang. Codes for cultivars: Fn39 = Funong 39; Fn38 = Funong 38; Yz06-407 = Yunzhe 06-407; Yz05-51 = Yunzhe 05-51; Lc03-1137 = Liucheng 03-1137; Gn02-70 = Gannan 02-70; and Roc22 = Roc22.

Upon pooling the yield data from all evaluation sites, the best performing cultivars can be identified visually by looking at a cultivars “point angle” in each artificial area from a GGE-Biplot in Figure 1. In general, cultivars located inside the polygon and near the origin are insensitive to environmental variations [27].

The cane yield GGE-Biplot constructed based on the 2011 plant cane test results was divided into five fan-shaped sectors (Figure 1(a)). The “point angle” cultivars were Gn02-70, Roc22, Yz06-407, Fn38, and Lc03-1137, clockwisely. Since there was no evaluation site within the first (top left) sector, Gn02-70, which was located at the “point angle”, had low cane yields in all tested sites. Yz06-407, followed by Fn38, had the best performances in cane yield since the evaluation Sites 1 (Guangxi Baise), 2 (Guangxi Chongzuo), 3 (Guangxi Hechi), 4 (Guangxi Laibin), 5 (Guangxi Liuzhou), and 6 (Yunnan Baoshan) fell into the 3rd sector. Similarly, Lc03-1137 in the 5th sector gave the best performance at evaluation Site 7 (Yunnan Lincang). Fn39 and Yz05-51 were not located at any “point angle” and therefore were insensitive to environmental variation.

Three fan-shaped sectors were formed in the cane yield GGE-Biplot obtained for the 2012 ratoon crop (Figure 1(b)). The “point angle” cultivars were Gn02-70, Yz06-407, and Lc03-1137. Again, Yz06-407 had the highest cane yield at the evaluation Sites 1, 2, 3, 4, 5, and 6. Gn02-70 and Roc22 gave poor cane yields in all evaluation sites. Lc03-1137 produced the highest cane yield at Site 7 (Yunnan Lincang), similar to the planted cane in 2011. Cultivars Fn39, Fn38, and Yz05-51 were insensitive to environmental variations.

#### 3.2.2. Sugar Yield

The sugar yield GGE-Biplot constructed based on the 2011 plant cane test results was divided into six fan-shaped sectors clockwisely (Figure 1(c)). The “point angle” cultivars were Yz06-407, Gn02-70, Yz05-51, LC03–1137, Fn38, and Roc22, respectively. Fn38, at the “point angle” of the 5th sector, had the highest sugar yield followed by Roc22 at the evaluation sites in Guangxi Baise, Guangxi Chongzuo, Guangxi Hechi, and Guangxi Laibin. Lc03-1137 had the highest sugar level in Guangxi Liuzhou (Site 7). GN02-70, followed by YZ 05-51, had the highest yield of sugar in this site of Yunnan Baoshan. Since the first sector did not contain any evaluation site, the “point angle” cultivar of Yz06-407 had low sugar yield at all test sites, while Fn39 was insensitive to environmental variation.

The sugar yield GGE-Biplot constructed based on the 2012 ratoon cropresults was divided into four sectors clockwisely (Figure 1(d)). The “point angle” cultivars were Gn02-70, Lc03-1137, Fn38, and Yz06-407, respectively. Since no test site was found in the first sector, Gn02-70, Roc22, and Fn39 produced low sugar yields in all evaluation sites. On the other hand, Fn38 had high sugar yield at the evaluation sites of Guangxi Baise (Site 1), Guangxi Chongzuo (Site 2), and Guangxi Hechi (Site 3). Lc03-1137, then Yz05-51, had the highest sugar yield in Guangxi Liuzhou (Site 5) and Yunnan Lincang (Site 7). Yz06-407 accumulated the highest sugar level at the evaluation sites Guangxi Laibin (Site 4) and Yunnan Baoshan (Site 6).

### 3.3. High Yielding Stability

#### 3.3.1. Cane Yield

**Figure 2:**
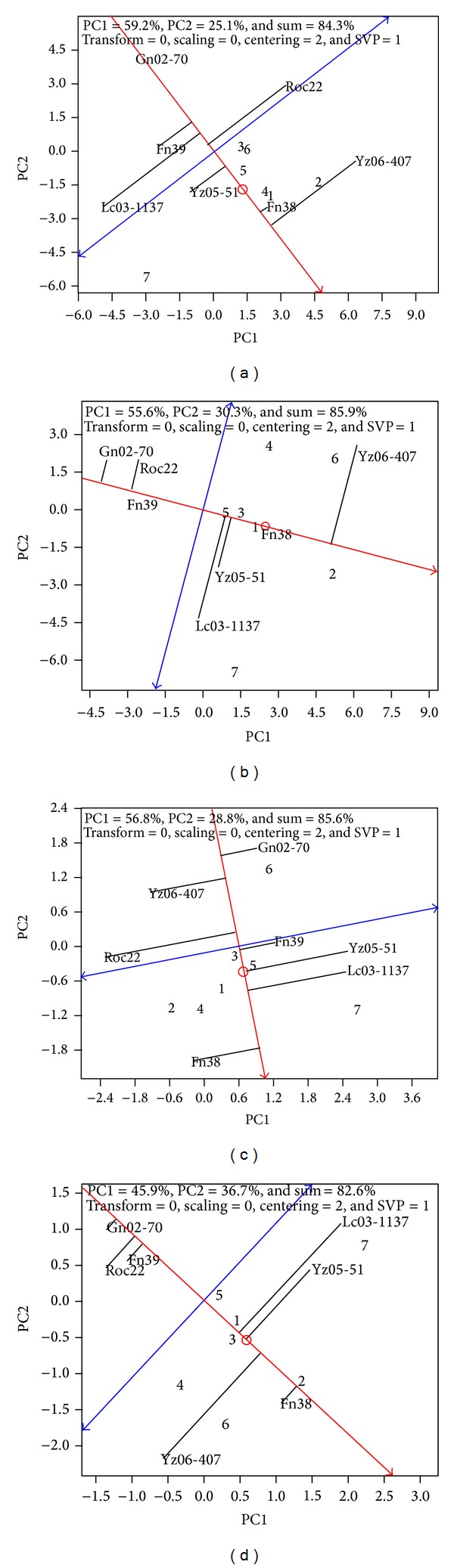
Stability of cane and sugar yields based on GGE-Biplot analysis. The circle represents the average environment. The single arrow red line is average environment-axis, projecting each cultivar's average yield from all evaluation sites. Through the original point and perpendicular to the red line, the double arrow blue line measures the vector stability between cultivar and environment. The closer to the average environment-axis, the more stable the cultivar's performance. Refer to Figure 1 legend for Panel, PC1, PC2, numerical codes for evaluation sites, and codes for cultivars.

Cultivars with high yielding potential across production years are ideal for sugarcane cultivation. This high yielding stability can be viewed directly on the GGE-Biplot shown in Figure 2. Main interaction between G and GE based on the 2011 plant cane crop (84.3%, Figure 2(a)) could be easily interpreted by the first principal component (PC1, 59.2%) and the second principal component (PC2, 25.1%). Among all cultivars tested, Yz06-407 had the highest cane yield, followed by Fn38 and Yz05-51. Lc03-1137, Fn39, and Gn02-70 had lower cane yield than that of the control Roc22. On the other hand, Fn38 and Gn02-70 had the highest stability, followed by Fn39 and Yz05-51. Yz06-407 and Lc03-1137 had lower stability than that of Roc22. Among all cultivars tested, Fn38 and Yz05-51 were the best in terms of both yield and stability, and Gn02-70 had the highest stability but the lowest yield, while Yz06-407 had the highest yield but the least stability.

Likewise, 85.9% interaction between G and GE based on the 2012 ratoon cane crop could be interpreted by PC1 (55.6%) and PC2 (30.3%) (Figure 2(b)). Again, Yz06-407 had the highest yield, followed by Fn38. Yz05-51 also had a relatively high yield. Lc03-1137, Fn39, and Gn02-70 had lower cane yields than that of Roc22. For yield stability, Fn38, Fn39, and Gn02-70 were the most stable, while Yz05-51, Yz06-407, and Lc03-1137 were less stable than Roc22. Among all cultivars evaluated, Fn38 was high in both cane yield and stability. Yz06-407 had the highest cane yield with low stability, while Gn02-70 had the lowest cane yield with high stability.

#### 3.3.2. Sugar Yield

Main part (85.6%) of *G* and GE interaction on 2011 plant cane sugar yield could be interpreted by PC1 (56.8%) and PC2 (28.8%) on the GGE-Biplot (Figure 2(c)). Fn38 had the highest cane yield, followed by Lc03-1137, Yz05-51, and Fn39. Yz06-407 and Gn02-70 had lower sugar yield than Roc22. Fn39, Gn02-70, Fn38, and Yz06-407 had highly stable sugar yield. The sugar yield stability of Lc03-1137 and Yz05-51 was lower than that of Roc22. Fn38 and Fn39 had the highest sugar yield with the highest stability. Gn02-70 was the most highly stable cultivar with low sugar yield. Yz05-51 and Lc03-1137 had high sugar yields with a poor stability.

For the sugar yield of 2012 ratoon crop, 82.6% of *G* and GE interaction could be figured out from the GGE-Biplot, which included 45.9% from PC1 and 36.7% from PC2 (Figure 2(d)). Just like for the plant cane crops, Fn38 topped the sugar yield list for its ratoon crop, followed by Yz05-51, Lc03-1137, and Yz06-407. Fn39 had a similar sugar level as Roc22, while Gn02-70 had a lower sugar yield than Roc22. Fn38, Fn39, and Gn02-70 showed the highest stability in sugar yield. Sugar yield stability was lower in Lc03-1137, Yz06-407, and Yz05-51 than in Roc22. Gn02-70 had the lowest sugar yield with relatively high stability, while Yz05-51, Lc03-1137, and Yz06-407 had high sugar yields but poor stabilities.

### 3.4. Representativeness and Discrimination Ability of Test Site

#### 3.4.1. Cane Yield

**Figure 3:**
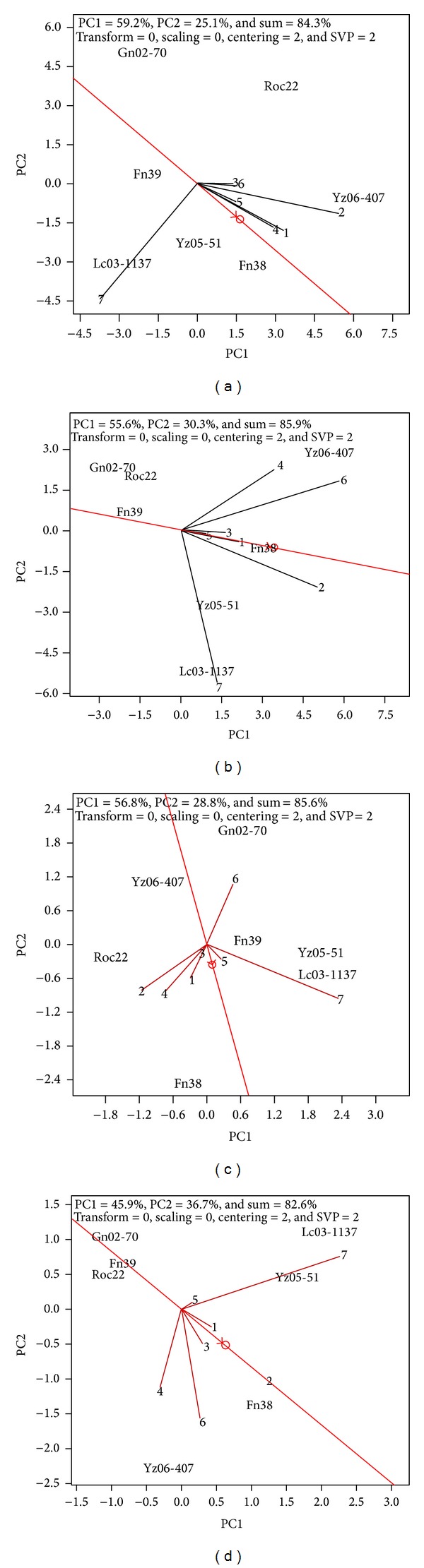
Representativeness and discrimination ability based on GGE-Biplot analysis. A line represents an environmental vector between original point and environment variance. An angle value between two lines (or vectors) represents the genetic correlation coefficient between the two. An angle value of <90° indicates a positive correlation; an angle value of >90° indicates a negative correlation; and an angle value of 90° indicates no correlation. Discrimination ability is measured by the line length of the environmental vector. The circle position depends on the average environmental value of all coordinate axes of the test sites; the single arrow red line connects the original point of bi-axis with average environmental value. Site representativeness is indicated by the angle between the single arrow red line and the site vector line. The smaller the angle, the stronger the representativeness. Refer to Figure 1 legend for Panel, PC1, PC2, numerical codes for evaluation sites, and codes for cultivars.

There was a relatively large GE effect on 2011 plant crop cane yield. There were positive correlations between each pair of sites among Guangxi Chongzuo, Guangxi Hechi, Yunnan Baoshan, Guangxi Baise, Guangxi Laibin, and Guangxi Liuzhou (Figure 3(a)). Comparing to other sites, Guangxi Baise, Guangxi Laibin, and Guangxi Liuzhou had better representativeness, and Yunnan Lincang, Guangxi Chongzuo, Guangxi Laibin, and Guangxi Baise had better discrimination ability. The discrimination ability of Guangxi Liuzhou, Guangxi Hechi, and Yunnan Baoshan was poor.

A relatively large GE effect was observed in 2012 ratoon crop trials. There were positive correlations between Yunnan Baoshan and Guangxi Liuzhou and between each pair of four test sites, namely, Guangxi Chongzuo, Guangxi Laibin, Guangxi Baise, and Guangxi Hechi (Figure 3(b)). These four sites also had better representativeness than the other three sites. On the other hand, Guangxi Chongzuo, Guangxi Laibin, Yunnan Lincang, and Yunnan Baoshan had better discrimination ability than Guangxi Hechi, Guangxi Liuzhou, and Guangxi Baise.

#### 3.4.2. Sugar Yield

There was no significant correlation between 2011 plant crop sugar yield traits when all test sites were taken into account. There were positive correlations between each pair of four test sites, namely, Guangxi Baise, Guangxi Laibin, Guangxi Chongzuo, and Guangxi Hechi and also between Guangxi Liuzhou and Yunnan Lincang (Figure 3(c)). Guangxi Baise, Guangxi Hechi, Guangxi Liuzhou, and Yunnan Lincang had better representativeness. A large vector angle between Yunnan Baoshan and the average environment indicated its poor representativeness on sugar yield. Guangxi Chongzuo, Guangxi Laibin, Guangxi Baise, Yunnan Baoshan, and Yunnan Lincang had better discrimination ability than the other two test sites, that is, Guangxi Liuzhou and Guangxi Hechi.

For the 2012 ratoon crop sugar yield traits, again no significant correlation was observed when all test sites were considered. There were strong positive correlations between Yunnan Lincang and Guangxi Liuzhou and between each pair of three test sites, namely, Guangxi Chongzuo, Guangxi Baise, and Guangxi Hechi (Figure 3(d)). These three sites had better representativeness than the other four sites. Guangxi Chongzuo, Guangxi Laibin, Yunnan Lincang, and Yunnan Baoshan had better discrimination ability than the other three sites.

## 4. Discussion and Conclusions

The two main objectives of sugarcane breeding are high cane yield and high sugar yield [1]. Traditional data analysis in sugarcane breeding often encounters difficulty to identify the cultivars that are both high yielding and adaptive to large, specific production areas. This is due to the complex sugarcane genome and unusual level of *G* × *E* effect. The GGE-Biplot software provides one of the most advanced statistical tools to circumvent this problem. The GGE-Biplot data is composed of genotype main effect *G* and genotype-environment interaction effect GE. It has been widely applied in Canada and the US to process yield and quality data [15]. Using GGE-Biplot, complex patterns of interactions between different factors can be revealed [14–17]. The stability of all cultivars can be demonstrated. In addition, representativeness and discrimination ability at different test sites can be disclosed [27].

Total sugar yield, a product of tonnage and sucrose content, is a final criterion to evaluate a sugarcane cultivar [25]. In this study, six new Chinese sugarcane cultivars were evaluated using the GGE-Biplot program. For both plant cane and first ratoon crop, cultivar Fn38 was the first in sugar yield and the second in cane yield. The cultivar is also stable in sugar production. Cultivar Gn02-70 had high stability but lower sugar yield. For both crops, Yz06-407 had a relatively high sugar yield due to its very high cane yield. However, this cultivar had poor stability. Yz05-51 and Lc03-1137 also showed high sugar yield with poor stability. Fn39, on the other hand, produced stable high sugar yield, although its cane yield was unstable, especially in the ratoon crops. It should be pointed out that all these six new sugarcane cultivars met with the National Sugarcane Qualification Standard [4] and were approved through the national sugarcane cultivar identification in July 2013.

Previous studies [15] revealed that GGE-Biplot might not fully show the rule of the data due to complex GE relationship and strong cultivar (*G*) effect. The analysis could be improved by running more than one round. For example, removal of low yielding cultivars from some or all test sites could reduce the weight of *G*, thereby enabling more GE to be distributed in the Biplot and a better discrimination of the environment [15]. For ratoon crops, the genotype-environment interaction is stronger than that of plant cane crop. In this study, a number of low yielding or nonpopular cultivars were removed, including Yuegan 35, Funong 36, Yuegan 34, Yunzhe 04-241, and another control ROC16. As a result, the GGE-Biplot analysis gave more accurate GE effects, representativeness, and discrimination ability of the remaining cultivars. The results met with the national standard on identification and evaluation of cultivars in high yield and its stability [25]. They also provided reference information to cultivar distributors, cane growers, and sugar mills for the selection of the most adaptable sugarcane cultivars.

The main objectives of sugarcane regional tests are to evaluate cultivars based on average performance from the whole region and to identify elite ones. Distribution and cultivar recommendation are also based on average performance on yield, quality, and disease resistance. Less attention has been paid to stability and adaptability of the cultivars across different sites and years. Genotypic adaptability to a specific production area has rarely been considered, except for the determination of suitable regions for released cultivars [25, 28]. Theoretically, an ideal sugarcane cultivar should be high yielding, stable yielding, and suitable for various environments. Our results demonstrate that Fn38 is one of such ideal sugarcane cultivars that is also resistant to mosaic, smut, and drought (unpublished). The only drawback was overtillering, which can be controlled by hilling in cultivation.

Regional distribution of a cultivar specifically suitable to corresponding ecological conditions is a strategy to increase large-scale sugarcane production, even though the general stability of that cultivar may be low across different regions. Using GGE-Biplot to identify cultivars that are adaptive to different regions may help achieve high yield and stability in each region [11]. For example, Yz05-51 and Lc03-1137 that had a poor adaptability across all the test sites also had high sugar yield with special adaptability to some sites, such as Guangxi Liuzhou (Site 5) and Yunnan Lincang (Site 7). Yz06-407 was another example, which had increased sucrose content at later harvesting season and could be planted with other later harvesting cultivars. In contrast, Gn02-70 had high and stable sucrose content at early stages and therefore could be harvested very early.

Sugarcane regional tests may also provide information about cultivar suitability to particular ecological zones. When a cultivar is recommended for production, its response to genotype-environment interaction should be considered. However, some cultivars may be missed in identification due to their average performance in large area and some neighboring sites [25]. The GGE-Biplot method can overcome this problem by displaying both high yielding ability and stability of cultivars over all test sites. Our results showed that Guangxi Chongzuo, Guangxi Baise, and Guangxi Hechi had a better representativeness in terms of both cane and sugar yield than that of other test sites. On the other hand, Guangxi Chongzuo, Yunnan Lincang, and Yunnan Baoshan had better discrimination ability than other test sites. Low discrimination ability at a test site could be due to environmental or human effect. Finally, removal of abnormal data can improve the reliability of GGE-Biplot analysis. For example, if all cultivars show low yield at a specific test site, either natural disasters or human factors may have been involved. As such, all data from this site should be removed from the GGE-Biplot analysis. It is therefore highly recommended that test sites be selected based on the following: (1) reference data collected over a longer period of time and (2) on-site investigations to reveal any natural disaster or human error [25].

In conclusion, cane and sugar yield and stability of six new sugarcane cultivars were presented. Furthermore, representativeness and discrimination ability of seven test sites were demonstrated. All varietal data revealed by GGE-Biplot provide a good reference for the identification of the cultivars suitable for multicultivar distribution for sugarcane production under different ecological environments.

## Figures and Tables

**Table 1:** Cane and sugar yields of sugarcane cultivars at seven test sites.

Traits	Season	Cultivar	GXBS	GXCZ	GXHC	GXLB	GXLZ	YNBS	YNLC	Mean	IR (%)
Cane yield (Mg·ha^−1^)	2011 plant	Roc22	97.64 ± 0.53^b^	150.44 ± 4.69^b^	80.14 ± 1.64^ab^	90.17 ± 11.12^a^	120.49 ± 12.78^a^	104.83 ± 1.04^d^	97.89 ± 17.74^d^	105.94 ± 23.43^c^	—
		Fn38	110.8 ± 1.36^a^	151.56 ± 5.44^b^	86.73 ± 12.83^a^	95.90 ± 6.86^a^	123.93 ± 1.17^a^	109 ± 4.36^cd^	131.32 ± 9.66^bc^	115.61 ± 22.91^ab ^	9.12
		Fn39	85.24 ± 0.37^e^	130.91 ± 7.62^cd^	68.17 ± 11.73^b^	82.50 ± 7.13^ab^	114.96 ± 4.11^a^	117.58 ± 10.69^bcd^	132.13 ± 1.47^bc^	104.50 ± 27.25^c^	−1.36
		Gn02-70	79.17 ± 3.52^f^	117.21 ± 4.33^d^	79.98 ± 13.26^ab^	65.77 ± 7.46^b^	113.06 ± 1.56^a^	119.27 ± 6.50^bc^	114.36 ± 8.37^cd^	98.40 ± 22.20^d^	−7.11
		Lc03-1137	93.19 ± 1.71^c^	120.76 ± 11.97^d^	73.07 ± 3.13^ab^	78.20 ± 9.68^ab^	116.88 ± 1.91^a^	106.44 ± 5.07^cd^	153.41 ± 7.86^a^	105.99 ± 26.93^c^	0.05
		Yz05-51	87.92 ± 2.49^d^	140.92 ± 9.59^bc^	78.69 ± 7.87^ab^	86.60 ± 16.21^a^	121.8 ± 1.77^a^	123.87 ± 7.77^b^	141.26 ± 9.85^ab^	111.58 ± 26.41^b^	5.32
		Yz06-407	109.51 ± 0.43^a^	168.2 ± 15.39^a^	83.26 ± 4.31^a^	94.50 ± 17.32^a^	129.48 ± 13.48^a^	139.08 ± 11.47^a^	111.04 ± 4.69^d^	119.30 ± 28.87^a^	12.61
		Sites mean	94.78 ± 11.50^e^	140.00 ± 20.91^a^	78.58 ± 9.58^g^	84.80 ± 13.87^f^	120.09 ± 10.97^c^	117.15 ± 13.00^d^	125.92 ± 20.93^b^		
	2012 ratoon	Roc22	130.80 ± 2.33^ab^	120.60 ± 1.52^d^	84.46 ± 7.19^ab^	81.33 ± 6.35^b^	92.27 ± 6.23^c^	79.06 ± 6.32^c^	83.80 ± 1.93^d^	96.05 ± 21.04^c^	—
		Fn38	127.07 ± 3.11^b^	151.10 ± 4.51^a^	100.27 ± 2.36^c^	85.33 ± 5.86^b^	96.97 ± 5.05^bc^	101.27 ± 7.32^b^	104.50 ± 8.75^c^	109.50 ± 22.12^b^	14.00
		Fn39	112.94 ± 3.99^c^	130.40 ± 4.00^c^	87.98 ± 3.94^ab^	77.67 ± 2.89^b^	99.27 ± 3.55^bc^	76.28 ± 4.02^c^	90.70 ± 4.58^d^	96.46 ± 20.34^c^	0.43
		Gn02-70	109.96 ± 3.53^c^	107.25 ± 3.50^e^	98.02 ± 5.86^ab^	66.33 ± 5.51^c^	101.23 ± 1.03^ab^	85.85 ± 4.75^c^	86.30 ± 0.52^d^	93.56 ± 17.13^c^	−2.59
		Lc03-1137	125.55 ± 5.36^b^	141.15 ± 1.96^b^	98.27 ± 0.96^bc^	60.67 ± 4.16^c^	103.40 ± 3.33^ab^	88.10 ± 4.57^c^	129.40 ± 5.91^a^	106.65 ± 27.55^b^	11.03
		Yz05-51	130.64 ± 2.49^ab^	144.45 ± 1.88^b^	91.19 ± 7.95^a^	84.33 ± 2.89^b^	99.10 ± 1.68^bc^	84.26 ± 9.13^c^	117.30 ± 5.27^b^	107.32 ± 23.79^b^	11.74
		Yz06-407	133.98 ± 3.65^a^	153.45 ± 5.26^a^	104.90 ± 1.13^ab^	102.67 ± 2.08^a^	108.30 ± 4.71^a^	131.83 ± 7.14^a^	91.70 ± 7.38^d^	118.12 ± 24.17^a^	22.98
		Sites mean	124.42 ± 9.34^b^	135.49 ± 16.34^a^	95.01 ± 8.08^cd^	79.76 ± 13.55^e^	100.08 ± 8.11^c^	92.38 ± 25.50^d^	100.53 ± 19.44^c^		

Sugar yield (Mg·ha^−1^)	2011 plant	Roc22	12.11 ± 0.07^c^	22.61 ± 0.71^ab^	10.37 ± 0.21^ab^	14.25 ± 1.76^ab^	16.83 ± 1.78^abc^	16.03 ± 0.16^d^	16.17 ± 1.20^e^	15.48 ± 3.83^b^	—
		Fn38	14.60 ± 0.18^a^	23.20 ± 3.67^a^	12.24 ± 1.81^a^	15.20 ± 1.09^a^	17.93 ± 0.17^a^	15.91 ± 0.64^d^	21.51 ± 1.58^bc^	17.23 ± 3.99^a^	11.29
		Fn39	11.90 ± 0.05^c^	19.45 ± 2.44^abc^	9.46 ± 1.63^b^	12.78 ± 1.10^abc^	16.84 ± 0.58^ab^	18.37 ± 0.33^bc^	21.90 ± 1.41^b^	15.81 ± 4.44^b^	2.16
		Gn02-70	11.15 ± 0.50^d^	18.42 ± 0.68^c^	11.75 ± 1.95^ab^	10.16 ± 1.16^c^	17.31 ± 0.24^ab^	20.32 ± 1.11^a^	19.14 ± 1.40^cd^	15.46 ± 4.17^b^	−0.10
		Lc03-1137	11.95 ± 0.22^c^	18.26 ± 1.81^c^	9.63 ± 0.41^b^	11.66 ± 1.44^bc^	16.06 ± 0.26^bc^	16.95 ± 0.81^cd^	24.96 ± 1.28^a^	15.64 ± 5.00^b^	1.02
		Yz05-51	11.94 ± 0.34^c^	18.84 ± 1.28^bc^	10.68 ± 1.07^ab^	12.46 ± 2.33^abc^	18.58 ± 0.27^a^	20.12 ± 1.27^a^	24.69 ± 1.72^a^	16.76 ± 5.04^a^	8.26
		Yz06-407	12.80 ± 0.05^b^	20.81 ± 1.90^abc^	10.01 ± 0.52^ab^	13.76 ± 2.52^ab^	15.20 ± 1.58^c^	19.74 ± 0.88^ab^	16.84 ± 0.71^de^	15.59 ± 3.83^b^	0.74
		Sites mean	12.35 ± 1.07^d^	20.23 ± 2.56^a^	10.59 ± 1.46^e^	12.90 ± 2.15^d^	17.01 ± 1.33^c^	18.21 ± 1.95^b^	20.74 ± 3.52^a^		
	2012 ratoon	Roc22	19.73 ± 0.35^a^	18.79 ± 0.24^a^	12.02 ± 1.03^d^	12.31 ± 0.96^b^	13.36 ± 0.90^a^	12.39 ± 1.34^c^	14.30 ± 0.33^c^	14.70 ± 3.13^c^	—
		Fn38	19.69 ± 0.48^a^	23.19 ± 0.69^a^	15.08 ± 0.36^a^	13.03 ± 0.90^ab^	14.56 ± 0.76^a^	16.64 ± 1.80^b^	17.95 ± 1.50^b^	17.16 ± 3.40^a^	16.75
		Fn39	17.20 ± 0.61^c^	19.37 ± 0.59^c^	12.58 ± 0.56^cd^	11.91 ± 0.44^b^	14.81 ± 1.82^a^	12.63 ± 0.67^c^	15.07 ± 1.03^c^	14.80 ± 2.71^c^	0.65
		Gn02-70	16.58 ± 0.53^c^	16.14 ± 0.53^c^	13.88 ± 0.83^abc^	9.96 ± 0.83^c^	14.67 ± 1.35^a^	14.44 ± 1.36^bc^	15.51 ± 1.47^c^	14.45 ± 2.27^c^	−1.67
		Lc03-1137	18.65 ± 0.80^b^	20.73 ± 0.29^b^	13.72 ± 0.13^bc^	8.96 ± 0.61^c^	14.80 ± 0.48^a^	14.60 ± 1.88^bc^	22.06 ± 2.05^a^	16.22 ± 4.42^b^	10.32
		Yz05-51	19.16 ± 0.37^ab^	21.94 ± 0.29^ab^	13.18 ± 1.15^bcd^	12.05 ± 0.41^b^	14.53 ± 0.49^a^	13.45 ± 1.33^c^	20.67 ± 0.93^a^	16.43 ± 3.89^b^	11.74
		Yz06-407	18.20 ± 0.50^b^	21.42 ± 0.74^b^	14.48 ± 0.16^ab^	14.12 ± 0.29^a^	14.04 ± 0.61^a^	19.18 ± 1.09^a^	14.50 ± 1.17^c^	16.56 ± 2.91^b^	12.67
		Sites mean	18.46 ± 1.24^b^	20.23 ± 2.26^a^	13.56 ± 1.17	11.76 ± 1.77^e^	14.39 ± 0.99^f^	14.76 ± 2.58^d^	17.15 ± 3.18^c^		

GXBS: Guangxi Baise; GXCZ: Guangxi Chongzuo; GXHC: Guangxi Hechi; GXLB: Guangxi Laibin; GXLZ: Guangxi Liuzhou; YNBS: Yunnan Baoshan; YNLC: Yunnan Lincang; IR: increasing rate; Fn39: Funong 39; Fn38: Funong 38; Yz06-407: Yunzhe 06-407; Yz05-51: Yunzhe 05-51; LC03-1137: Liucheng 03-1137; Gn02-70: Gannan 02-70; Roc22: Roc22. Different letter means significance at the level of 0.05.

**Table 2:** Analysis of variance on cane and sugar yields of sugarcane cultivars.

Variance source	Cane yield						Sugar yield					
	2011 plant			2012 ratoon			2011 plant			2012 ratoon		
	SS	*F*	*P*/%	SS	*F*	*P*/%	SS	*F*	*P*/%	SS	*F*	*P*/%
Treatment	229983.68	25.03**		214770.99	13.78**		6544.98	30.43**		4887.33	12.58**	
Genotype	25816.79	14.80**	11.23	30447.69	15.65**	14.18	700.84	5.93**	10.71	877.83	9.28**	17.96
Environment	151579.98	151.87**	65.91	127088.65	73.51**	59.17	4627.79	197.73**	70.71	2612.82	69.83**	53.46
Interaction	52586.91	5.60**	22.87	57234.64	3.51**	26.65	1216.35	6.62**	18.58	1396.68	3.59**	28.58

*P < 5%; **P < 1%.
